# Adiponectin is associated with inflammaging and age-related salivary gland lipid accumulation

**DOI:** 10.18632/aging.204618

**Published:** 2023-03-27

**Authors:** Ji Won Kim, Jeong Mi Kim, Mi Eun Choi, Eun Jeong Jeon, Jin-Mi Park, Young-Mo Kim, Jeong-Seok Choi

**Affiliations:** 1 Department of Otorhinolaryngology-Head and Neck Surgery, Inha University College of Medicine, Jung-gu, Incheon 22332, Republic of Korea; 2 Department of Biomedical Science, Program in Biomedical Science and Engineering, Inha University, Michuholgu, Incheon 22212, Republic of Korea; 3 Research Center for Controlling Intercellular Communication (RCIC), College of Medicine, Inha University, Michuholgu, Incheon 22212, Republic of Korea

**Keywords:** adiponectin, inflammation, aging, salivary gland, lipid

## Abstract

Dry mouth is frequently observed in the elderly, and enhanced lipid accumulation plays a critical role in cellular senescence in the salivary gland (SG). We investigated the mechanisms that mediate lipogenesis-associated SG senescence. Adult (28.6 ± 6.6 y.o. and 43.3 ± 1.5 y.o.) and aged (82.0 ± 4.3 y.o. and 88.0 ± 4.3 y.o.) human parotid and submandibular glands were compared with respect to histologic findings, 8-OHdG (8-hydroxy 2 deoxyguanosine) expression patterns, TUNEL (Terminal deoxynucleotidyl transferase dUTP nick end labeling) and SA-β-gal (senescence-associated β-galactosidase) assay results. Also, microarray analysis was performed on RNA extracted from adult and aged SG to identify DEGs (differentially expressed genes). The effects of silencing ADIPOQ (Adiponectin) were evaluated by quantifying cell proliferation, immunohistochemical staining for cellular senescence and inflammation-associated proteins, SA-β-gal assays, RT-PCR, and western blot. Histological findings demonstrated the presence of more lipocytes, chronic inflammation, fibrosis, and lymphocytic infiltration in old SG. In addition, old tissues demonstrated higher expressions of SA-β-gal, more apoptotic cells in TUNEL assays, and higher oxidative stress by 8-OHdG immunostaining. Microarray analysis showed lipogenesis was significantly upregulated in old tissues. Silencing of ADIPOQ (a lipogenesis-related gene) reduced inflammation and SA-β-gal levels and increased cell proliferation and the expressions of amylase and aquaporin 5 in human SG epithelial cells. The study shows ADIPOQ is a potential target molecule for the modulation of lipogenesis associated with SG senescence.

## INTRODUCTION

Saliva play crucial roles during chewing, swallowing, and tasting, and has an important antibacterial effect. As we age, the amount of saliva produced decreases, which is often considered an aging-associated phenomenon. Aging is defined as a time-dependent reduction in the physiologic functions of organs [1], and the elderly commonly exhibit salivary dysfunction resulting in dry mouth [2]. Age-related histological changes in salivary glands (SGs) include acinar atrophy, fibrosis, focal lymphocytic infiltration, ductal changes (e.g., hyperplasia and dilatation [3]), a decrease in the mean volume fraction of acini, an increase in the volumes of vessels and connective and adipose tissues, and an increase of inflammatory infiltration [4]. Fatty infiltration is a characteristic of Sjögren’s syndrome, though aging is similarly associated with fat replacement [5]. Furthermore, it was suggested in an animal experiment that lipofuscin accumulation and secretory granule degeneration might be related to reduced SG cellular secretory activity in older rats [6].

Few reports have been issued on the mechanisms responsible for the age-related lipid infiltration of SGs or age-related gene expressional differences in the SG tissues of young and old adults. Herein, we investigated the following: (1) the mechanisms involved in inflammaging of SGs, (2) the differential expressions of genes in adult and aged SGs, and (3) the molecules primarily responsible for cellular senescence of SG.

## RESULTS

### The proportion of lipocytes in SGs increased with age

**Figure 1:**
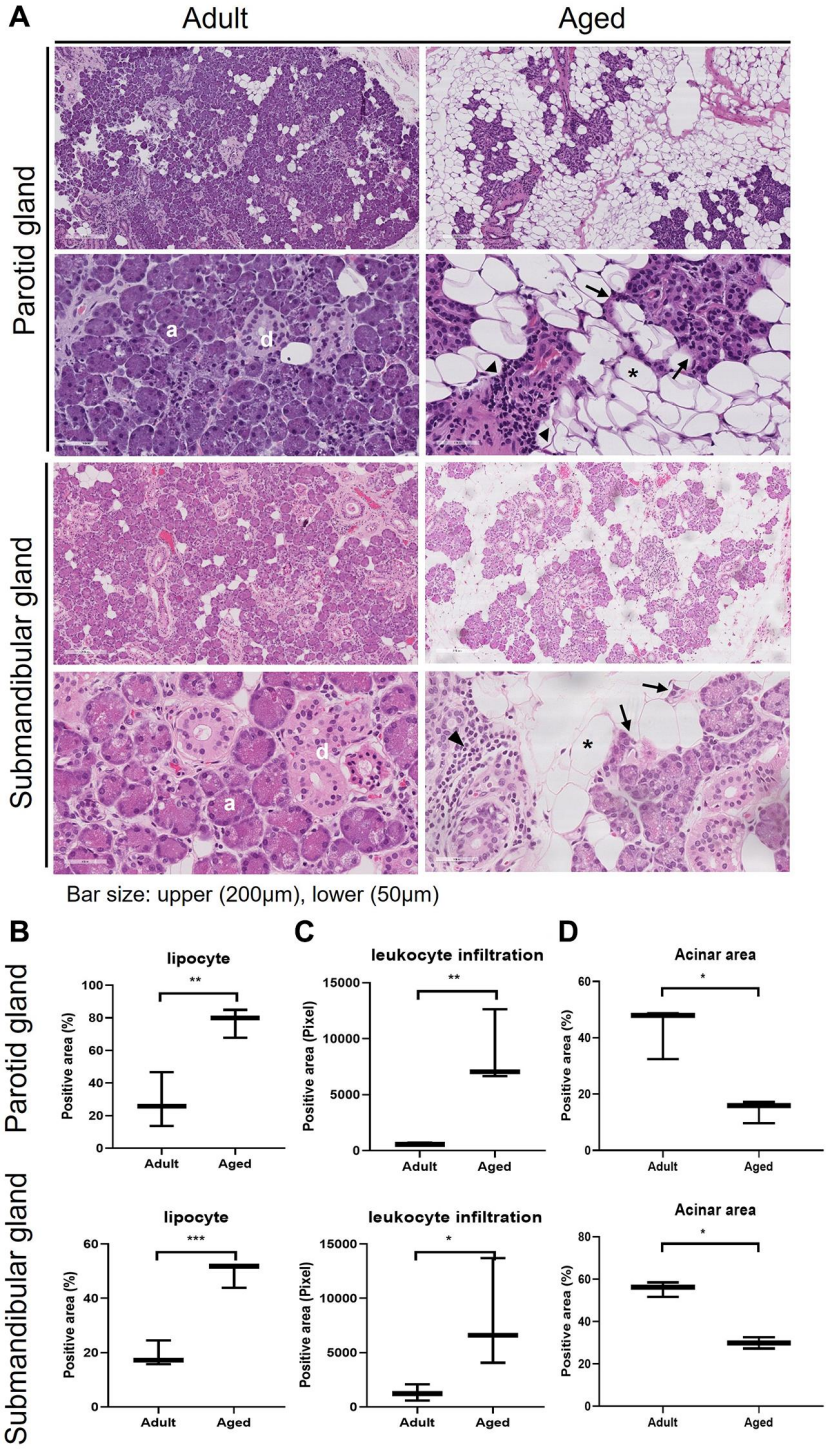
**Hematoxylin and eosin (H&E) stained tissue sections showing age-related morphological changes in human parotid and submandibular glands.** (**A**) The morphology of adult and aged salivary glands, bar size: upper (200 um), lower (50 um). (**B**–**D**) The quantifications of lipocyte, leukocyte infiltration and acinar area. Results are presented as the mean ± SD and *T*-test was performed for statistical analysis ^**^p < 0.01, ^*^p < 0.05.

We compared SG morphologies in the human parotid gland (PG) and submandibular gland (SMG) tissues of 3 adult (28.6 ± 6.6 y.o. in PG and 43.3 ± 1.5 y.o. in SMG) and aged (82.0 ± 4.3 y.o. in PG and 88.0 ± 4.3 y.o. in SMG) subjects. Hematoxylin and eosin (H&E) stained images of the human PG and SMG are shown in Figure 1A and the positive area of adipocyte, leukocyte infiltration, and acinar cells are quantified in Figure 1B–1D, respectively. Age-related SG change was assessed by determining the proportion of lipid droplets. The histological findings in old SGs demonstrated the presence of more lipocytes, chronic inflammation, fibrosis, and foci of leukocyte infiltration. Adult SGs exhibited a dense packed lobular acinar structure and well-organized ductal alignment, whereas aged SGs exhibited atrophied acinar cells and lipocyte and leukocyte infiltration.

### Age-related SG morphologic changes were associated with apoptosis and oxidative stress

**Figure 2:**
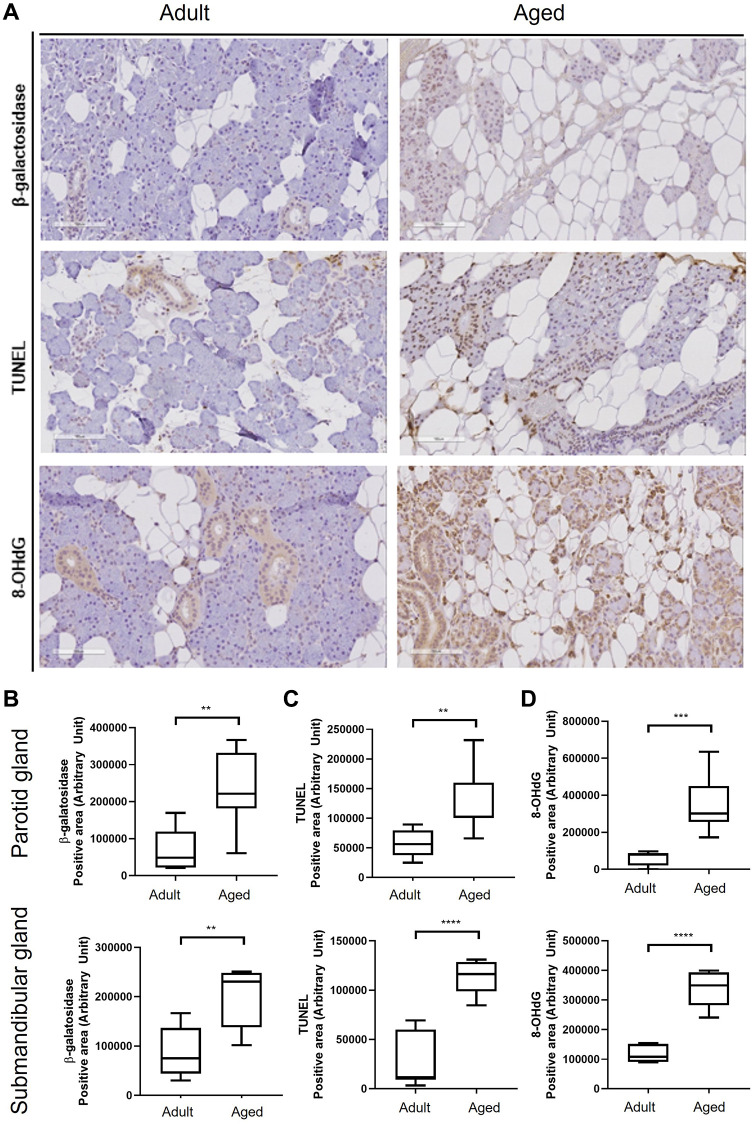
**Cellular senescence in human salivary gland tissue.** (**A**) β-Galactosidase assay, TUNEL assay, and the expression of 8-OHdG. (**B**) Quantitation of β-galactosidase positive areas. (**C**) Quantitative analysis of TUNEL (terminal deoxynucleotidyl transferase dUTP nick end labeling) positive areas. (**D**) Quantitative analysis of 8-OHdG (8-hydroxy-2' -deoxyguanosine) positive areas. Results are presented as the mean ± SD and *T*-test was performed for statistical analysis ^****^p < 0.0001, ^***^p < 0.001, ^**^p < 0.01.

During aging, substantial acinar cell loss occurs in SGs, and lipocytes accumulate in areas of loss. We found significant acinar cell loss and increases in 8-hydroxy-2′-deoxyguanosine (8-OHdG, a critical biomarker of oxidative stress) expression and apoptosis in aged SG tissues (Figure 2A), and more cellular senescence β-galactosidase (SA-β-gal) expression in aged PG and SMG tissues. Quantitative analysis also showed that the proportion of SA-β-gal positive senescent cells increased with age (*P* < 0.01, Figure 2B). TUNEL (terminal deoxynucleotidyl transferase dUTP nick end labeling) assays demonstrated old SG tissues contained more apoptotic cells than young tissues (*P* < 0.05, Figure 2C). Furthermore, immunohistochemical (IHC) staining for 8-OHdG was significantly greater in old SG tissues (*P* < 0.005, Figure 2D).

### Differentially expressed genes (DEGs) in young and old SG tissues

To determine whether aging alters gene expressions, we subjected young and old SG tissues to microarray analysis. Fifteen DEGs in PG and 31 in SMG exhibited absolute fold changes of > 2 (*P* < 0.05, respectively). In aged PG tissues, 7 of the 15 DEGs were upregulated and 8 were downregulated (*P* < 0.05), and in aged SMG tissues, 21 of the 31 were upregulated and 10 were downregulated. The top 10 DEGs are listed in Table 1. The functions of these DEGs were determined by Gene Ontology analysis and a comprehensive literature review [7–22]. All DEGs (fold changes of > 1.5, *p* < 0.05) in PG and SMG were listed in Supplementary Figures 1 and 2.

**Table 1:** Results for differentially expressed genes.

	**Gene**	**Regulation**	**Fold change (old vs. young)**	***P* value**	**Functions**
**SMG**	CSN3	Up	3.877379	0.0029	Neural differentiation [9]
	ODAM	Up	3.621289	0.0246	Cell adhesion [10]
	ZCWPW2	Up	3.485096	0.0033	−
	ADIPOQ	Up	3.281488	0.0358	Lipid metabolism, insulin sensitivity [17], autophagy [16]
	PLIN1	Up	2.753045	0.0409	Inflammation, lipid metabolism [14]
	TRDJ4	Up	2.563580	0.0135	−
	PEG10	Up	2.513301	0.0212	Carcinogenesis [13]
	DMBT1	Up	2.477414	0.0082	Cell growth [12], inflammation, innate immune process [11]
	LCN2	Up	2.469396	0.0076	Apoptosis, iron uptake [7]
	RPL23AP7	Up	2.445967	0.0277	−
**PG**	IGHM	Up	6.968879	0.0158	Immune response [22]
	LOC101927120	Up	2.908335	0.0427	−
	SLC7A5	Up	2.841554	0.0086	Transporter, inflammation [18]
	KRTAP5-2	Up	2.203520	0.0204	−
	SNORD37	Up	2.157255	0.0130	−
	KRT6B	Up	2.069454	0.0158	Apoptosis [19], cell migration [15]
	SERPINA5	Up	2.015452	0.0048	blood coagulation [8], spermatogenesis [20]
	CES1P1	down	2.259019	0.0437	−
	RNU12	down	2.211497	0.0095	−
	SCD	down	2.145316	0.0435	Lipid metabolism [21]

### KEGG pathway analysis of DEGs

**Figure 3:**
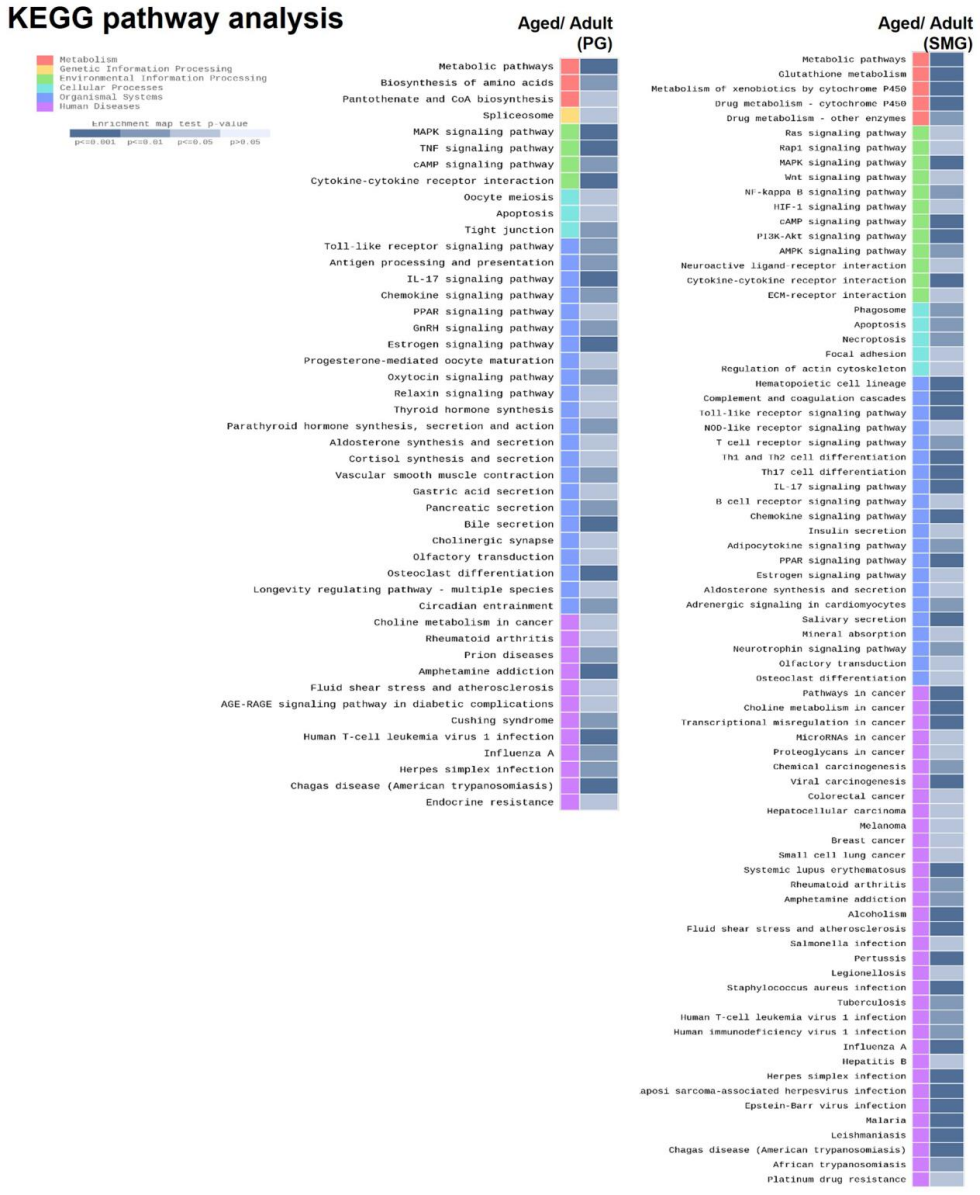
KEGG pathway analysis of DEGs derived from comparison of adult and aged salivary glands.

To explore the molecular mechanism of salivary gland aging, we performed enrichment analysis using Kyoto Encyclopedia of Genes and Genomes (KEGG) database. KEGG is a comprehensive database that integrates genomic, chemical, and systemic functional information better to understand the molecular response networking of coding genes. The data showed that these DEGs of aged PG were enriched in metabolic pathway, MAPK signaling pathway, Oocyte meiosis, Toll-like receptor signaling pathway, And the DEGs of aged SMG were enriched in metabolic pathway, RAS signaling pathway, phagosome, hematopoietic cell lineage, rheumatoid arthritis (Figure 3).

### ADIPOQ was associated with SG aging

**Figure 4:**
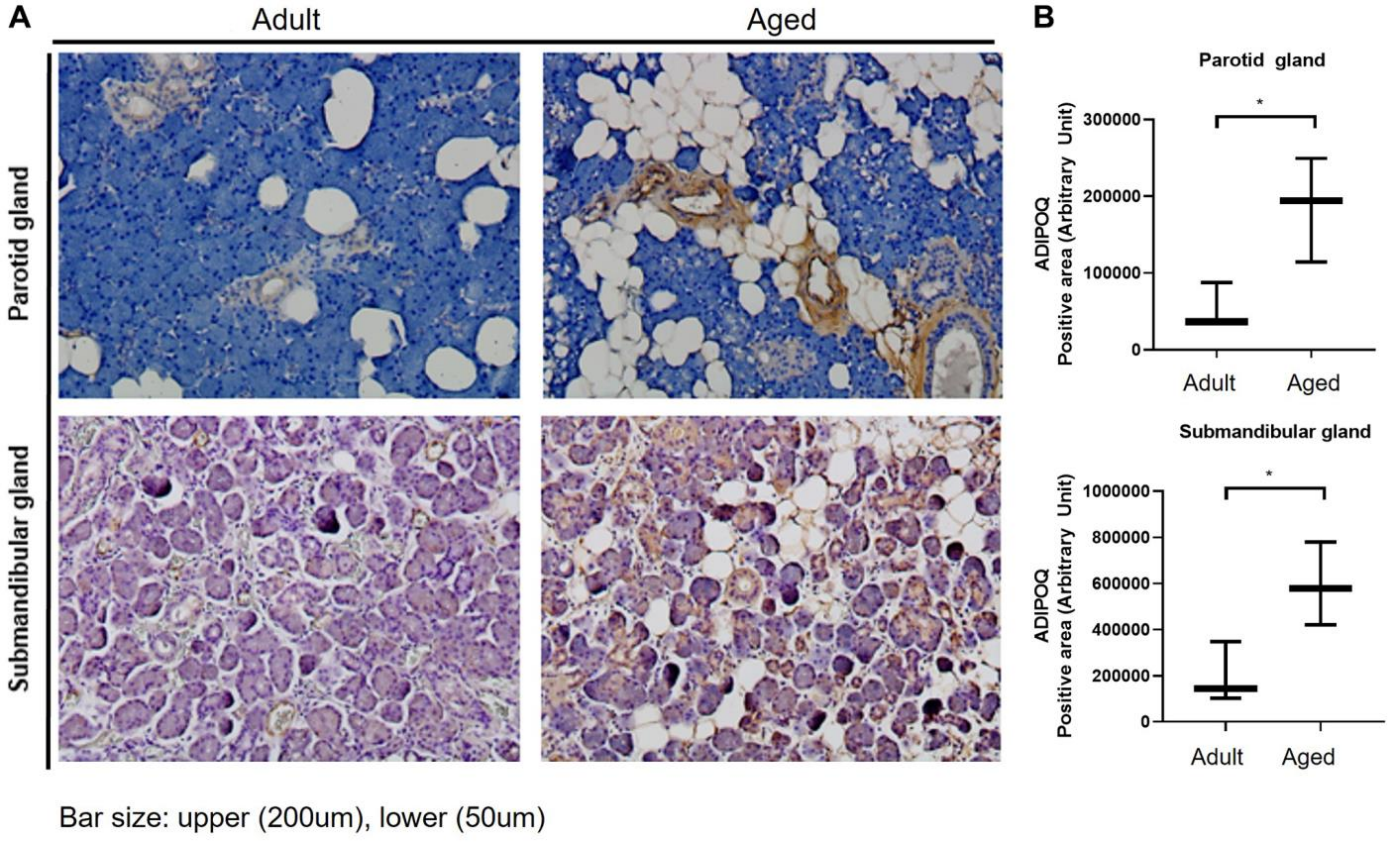
**Immunohistochemical (IHC) staining of ADIPOQ in adult and aged human salivary gland tissues.** (**A**) IHC staining of ADIPOQ in adult and aged parotid and submandibular gland tissues (**B**) Quantitative analysis of ADIPOQ expression in adult and aged parotid and submandibular gland tissues Results are presented as the mean ± SD and *T*-test was performed for statistical analysis ^*^p < 0.05.

ADIPOQ is commonly expressed in submandibular glands, and IHC staining showed it was upregulated in aged PG and SMG (Figure 4A). Quantitative analysis also showed ADIPOQ was significantly upregulated in aged SG tissues (*P* < 0.05, Figure 4B).

### ADIPOQ regulated cellular senescence

**Figure 5:**
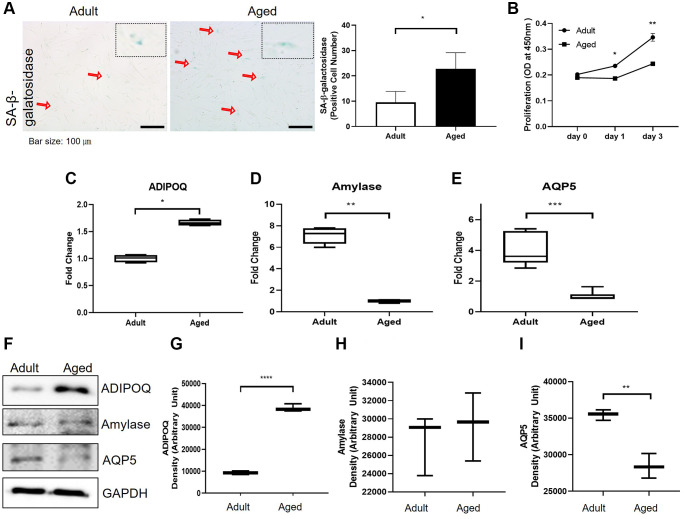
**Cellular senescence and the expressions of aging-related functional proteins in primary cultured salivary gland (SG) cells.** (**A**) β-Galactosidase stained adult and aged primary cultured SG cells. (**B**) The proliferation rate of adult and aged human SG cells. (**C**–**E**) The mRNA expressions of ADIPOQ, amylase, and AQP5 in adult and aged human parotid and submandibular gland tissues. (**F**–**I**) The protein expressions of ADIPOQ, amylase, and AQP5 in adult and aged human parotid and submandibular gland tissues. Results are presented as the mean ± SD and *T*-test was performed for statistical analysis, ^***^p < 0.001, ^**^p < 0.01, ^*^p < 0.05.

To examine SG cell senescence, we compared the expressions of SG functional proteins in primary cultured cells isolated from adult and aged healthy SG tissues. Significantly more SA-β-gal positive cells were detected in primary cells cultured from aged SG tissues (*P* < 0.05, Figure 5A). And the proliferation rate of the aged cells was significantly lower than that of the adult cells as shown in Figure 5B. Also, Western blot and RT-PCR showed aged SG cells expressed significantly more ADIPOQ but less aquaporin 5 than adult SG cells (Figure 5C, 5E, 5G, 5I and 5F). The mRNA level of amylase was decreased in aged SG cells, but the protein level of amylase was similar in adult and aged SG cells (Figure 5D, 5H and 5F). Uncropped western blot images were provided as Supplementary Figure 3.

### Silencing of ADIPOQ increased cell proliferation and suppressed SG cell dysfunction and proinflammatory reactions

**Figure 6:**
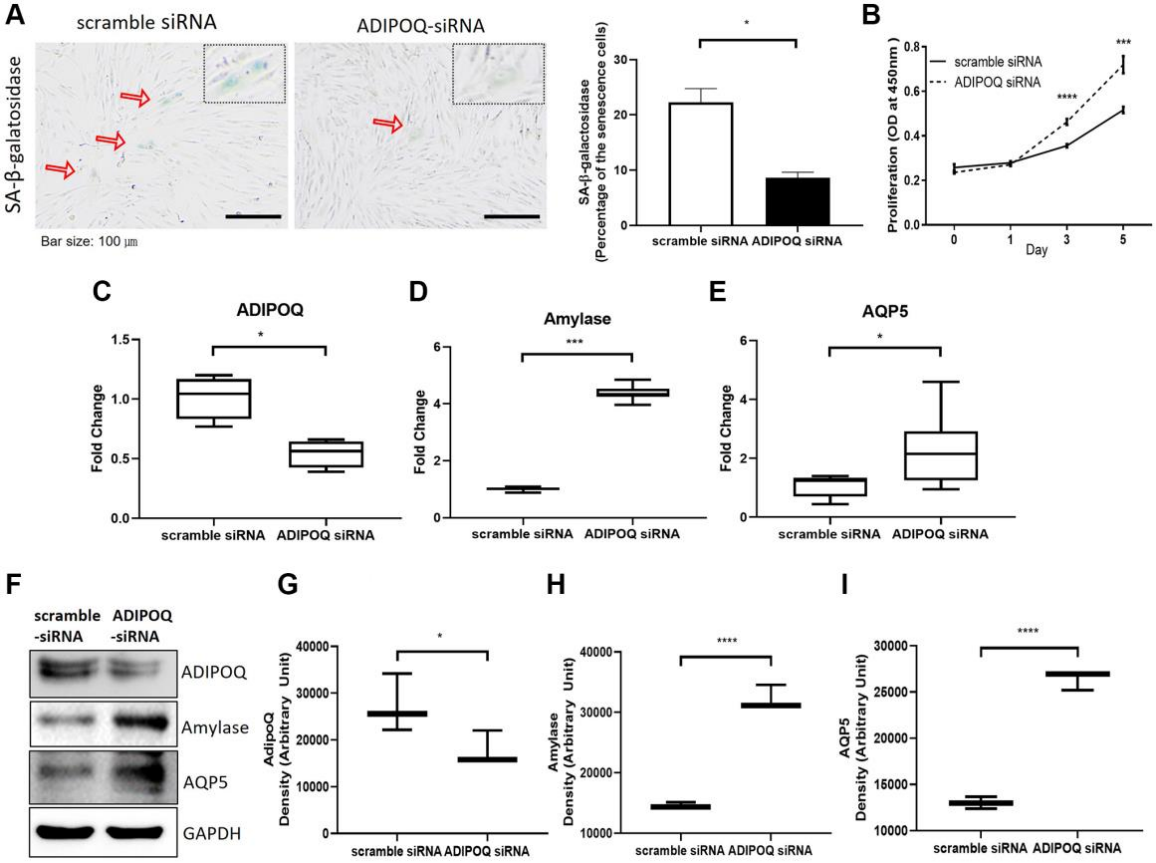
**Silencing of ADIPOQ reduced cellular senescence and enhanced the proliferation and expression of salivary functional proteins.** (**A**) β-Galactosidase assays of scrambled-siRNA and ADIPOQ-siRNA transfected primary cultured SG cells. (**B**) Proliferations of ADIPOQ-siRNA and scrambled siRNA treated SG cells. (**C**–**E**) The mRNA expressions of ADIPOQ, amylase, and AQP5 in ADIPOQ-siRNA and scramble-siRNA treated SG cells. (**F**–**I**) The protein levels of ADIPOQ, amylase and AQP5 in ADIPOQ-siRNA and scramble-siRNA treated SG cells. Results are presented as the mean ± SD and *T*-test was performed for statistical analysis, ^****^p < 0.0001, ^***^p < 0.001, ^*^p < 0.05.

To examine the functional role of ADIPOQ, we silenced its expression in human SG epithelial cells using siRNA (Figure 6C and 6G, *P* < 0.05, Figure 6F). ADIPOQ siRNA-treated aged SG cells exhibited significantly fewer SA-β-gal positive cells than adult cells treated with scrambled siRNA (*p* < 0.05, Figure 6A). Furthermore, ADIPOQ siRNA-treated SG cells proliferated significantly more than scrambled siRNA-treated SG cells (*P* < 0.0001 and *P* < 0.001, Figure 6B). We also studied the expressions of the SG functional molecules amylase and aquaporin 5 by Western blot and qRT-PCR and found the expressions of both were higher in ADIPOQ siRNA treated SG cells than in scrambled siRNA treated SG cells (Figure 6D, 6E, 6H and 6I, *P* < 0.001).

**Figure 7:**
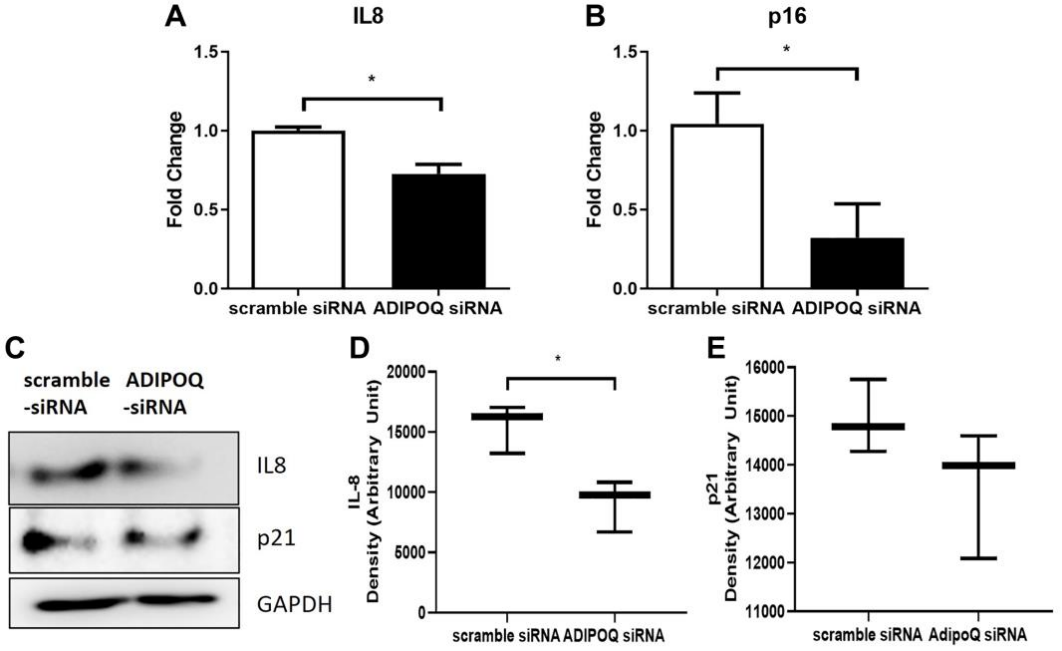
**Effects of ADIPOQ gene knockdown on inflammatory factors.** (**A**, **B**) Expressions of IL8 and p16 (proinflammatory genes) in ADIPOQ silenced cells. (**C**–**E**) Expressions of IL8 and p21 (a proinflammatory protein) in ADIPOQ silenced cells. Results are presented as the mean ± SD and *T*-test was performed for statistical analysis ^*^p < 0.05.

RT-PCR showed that IL-8 and p16 mRNA levels in SG primary cultured cells were significantly lower in ADIPOQ siRNA transfected cells than in scrambled siRNA transfected cells (*P* < 0.05, Figure 7A and 7B). The IL-8 protein expression level was significantly lower in ADIPOQ siRNA transfected cells than in scrambled siRNA transfected cells (Figure 7D, P < 0.05 and Figure 7C). Furthermore, ADIPOQ knockdown tended to reduce the expression of p21 in human SMG primary cultured cells (Figure 7E and *P* = 0.09, Figure 7C). Uncropped western blot images were provided as Supplementary Figures 4 and 5.

## DISCUSSION

Dry mouth leads to swallowing difficulties, oral cavity infections, and poor quality of life and is strongly associated with SG dysfunction, and aging is associated with secretory acinar cell dysfunction and lipid accumulation in SGs. Although several reports have been issued on age-induced lipid accumulation in SGs [3–5, 22], no study has investigated the mechanisms and effects of age-related lipocyte infiltration-related genes in SGs. In this study, we aimed to identify age-related morphologic changes in SGs and the mechanisms responsible. In addition, we identified age-related DEGs in the aged SG tissues and confirmed the effects of ADIPOQ on aging-related SG fatty replacement.

We previously showed that in mice, age-related SG changes are accompanied by reductions in saliva production and excretion [2]. Regarding morphologies, the SGs of old mice exhibited atrophied acinar cells, fibrotic ductal cells, and lymphocyte infiltration [2]. Syrjanen et al. demonstrated that the adipose tissues of the labial SGs of healthy adults increase significantly with age [23], and Dayan et al., in a human palatal SG histomorphometric study, reported age-induced increases in inflammatory infiltrates in blood, lymph vessels, and adipose and connective tissues [4]. In the present study, the proportions of fibrotic periductal and atrophied acinar cells and lipocytes were found to be greater in old SG tissues, which is consistent with previous results.

Age-associated acinar cell atrophy and leukocyte infiltration in SGs are considered results of chronic inflammation and increased apoptosis [2, 24], and we also observed a more significant proportion of TUNEL-positive cells in aged SG tissues. Furthermore, oxidative stress caused by ROS is associated with inflammation [25], and Knas et al. revealed a connection between oxidative stress and SG dysfunction in diabetic SG tissues [26]. In another study, accumulated lipocytes in the parenchyma of SGs released monocyte chemoattractant protein-1, and thus, promoted the transformation of monocytes to macrophages [27], which release proinflammatory cytokines, promote inflammation, and are capable of producing large amounts of ROS [26]. Also, age-related SG lipid infiltration may be similar mechanistically to fatty liver-induced cellular damage [25]. Lipid accumulation impairs the oxidative capacity of mitochondria, produces excessive ROS, and causes SG morphological changes [28]. In the present study, we found levels of the oxidative stress marker 8-OHdG were significantly higher in aged SG tissues, suggesting oxidative stress may be age-associated.

A comparison of the microarray data of adult and aged PG and SMG tissues revealed 15 and 31 DEGs, respectively. We focused on one of these genes, that is, ADIPOQ, because of its relevance in lipid accumulation. Adiponectin is a multifunctional hormone secreted by adipose tissues [29]. In particular, it regulates energy metabolism and inflammation [30–33] and is also involved in the regulation of apoptosis in various cell types [34]. However, few studies have examined the role of adiponectin in SG cells. Ding et al. suggested adiponectin promotes SG secretion by modulating tight junctions and that it also functions as a promoter of salivary secretion in rat submandibular glands by activating adiponectin receptors (AdipoRs) via AMPK (adenosine monophosphate activated protein kinase) activation [29]. A few studies have reported adiponectin and AdipoRs are expressed in salivary gland tissues [35, 36] and in those patients with Sjogren’s syndrome, minor salivary gland epithelial cells secrete more adiponectin than normal, which suggests that adiponectin and AdipoRs expressed in SGs can regulate inflammatory reactions [36, 37]. We checked the expression of AdipoR1 on human PG and SMG, and the AdipoR1 was strongly expressed in the duct of the salivary gland tissues shown in Supplementary Figure 6, suggesting the possibility that ADIPOQ bind to AdipoR1 in ductal cells to modulate several aging-related biological functions. Adiponectin is known to have pro-inflammatory and anti-inflammatory effects and has been reported to plFay proinflammatory roles in chronic inflammatory and autoimmune diseases such as rheumatoid arthritis, chronic kidney disease, and inflammatory bowel disease [38]. Miyagi et al. reported hyposalivation in the submandibular glands of aged mice and showed that this involved cell senescence, lymphocyte infiltration-related chronic inflammation, and reduced aquaporin 5 expression [39]. These findings are consistent with the concept of “inflammaging” meaning that lymphocyte deposition-related chronic inflammation is associated with aging [40]. In this study, we found that when ADIPOQ siRNA was administered to human SG cells, they proliferated more, proportions of senescent cells were lower than in non-treated controls, and amylase, aquaporin 5, and proinflammatory marker levels normalized. These observations suggest adiponectin might modulate age-associated chronic inflammation.

There is some limitation in this study. The number of samples are a little bit small. Extraction of the samples from more than 80 years old is not easy, and some cases are excluded due to failure of RNA QC. Further research is needed to prospectively validate our data.

In conclusion, our histologic study of chronic inflammation and lipid infiltration ratios were elevated in aged SG tissues. We tentatively suggest that the mechanisms responsible for these morphological changes are apoptosis and oxidative stress. Interestingly, adiponectin was identified as an age-related DEG in old SG tissues by microarray analysis. Also, the observation that ADIPOQ knockdown by siRNA transfection increased the expressions of amylase and AQP5 implies adiponectin is involved in SG aging through lipogenesis. These findings indicate adiponectin is a potential target for the treatment of age-related SG dysfunction and chronic inflammation.

## MATERIALS AND METHODS

### Preparation of human parotid and submandibular gland tissue specimens for cDNA microarray analysis

Human parotid gland and human submandibular gland tissues were obtained from patients that underwent parotidectomy for a benign parotid tumor. Tissue samples were examined by a pathologist, who excised normal tissues for cDNA microarray analysis. Human parotid gland and submandibular gland tissue samples from adult (28.6 ± 6.6 y.o. and 43.3 ± 1.5 y.o., respectively) and aged **(**82.0 ± 4.3 y.o. and 88.0 ± 4.3 y.o., respectively) subjects were examined and processed (*n* = 3, each). For the histopathological study, excised human parotid gland and human submandibular gland tissues were fixed in a 4% paraformaldehyde phosphate buffer solution, embedded in paraffin, and stained with H&E. In addition, clinical data (age, sex, and BMI) were retrospectively reviewed and summarized in Supplementary Table 1. All specimens were collected after obtaining informed consent and institutional review board approval **(**INHA 18 0503-560**)**.

### cDNA microarray analysis

Total RNAs from adult and aged SG tissues were extracted using the RNeasy Mini kit (Qiagen, Germany). RNA purity (The values of 1.7–2.2 of 260/230 and 260/280 ratio) and integrity (RIN >7) were confirmed using an ND-1000 Spectrophotometer (NanoDrop Technologies, Inc., Wilmington, DE, USA) and an Agilent 2100 Bioanalyzer (Agilent Technologies, Palo Alto, CA, USA). The Affymetrix Whole Transcript Expression array process was performed according to the manufacturer’s instructions (GeneChip Whole Transcript PLUS reagent Kit). cDNA was synthesized using the GeneChip WT (Whole Transcript) Amplification kit. Sense cDNA was fragmented and biotin-labeled with TdT (terminal deoxynucleotidyl transferase) using the GeneChip WT Terminal labeling kit. Approximately 5.5 μg of labeled target DNA was hybridized to the Affymetrix GeneChip Human 2.0 Array for 16 hours at 45°C. Hybridized arrays were then washed and stained on a GeneChip Fluidics Station 450 and scanned using a GCS3000 Scanner (Affymetrix). Signal values were computed using Affymetrix^®^ GeneChip^™^ Command Console software. Data were summarized and normalized using the robust multi-average (RMA) method implemented in Affymetrix^®^ Power Tools (APT). DEG analysis was performed using gene-level RNA analysis results. Statistical significances of expressional differences were determined using the independent *t*-test. The false discovery rate (FDR) was controlled by adjusting *p*-values using the Benjamini-Hochberg algorithm. To produce a DEG set, Hierarchical cluster analysis was performed using complete linkage and Euclidean distance as a measure of similarity. Gene-Enrichment and Functional Annotation analyses for the DEGs list using Gene Ontology (http://geneontology.org) and KEGG (http://kegg.jp). Data analysis and DEG visualization were conducted using R 3.3.3 (http://www.r-project.org).

### Histological analysis and immunohistochemistry

Human PG and SMG tissue sections were dewaxed, hydrated, stained with H&E, and examined under a digital microscope (Olympus, Japan). The IHC study was performed as previously described [41] using antibodies for ADIPOQ, b-galactosidase, and 8-OHdG (1:1000; Santa Cruz, CA, USA). A blinded examiner evaluated three random fields per section, and stained areas were measured in pixels using Image J software (MD Anderson Cancer Center, TX, USA).

### Terminal deoxynucleotidyl transferase dUTP nick end labeling (TUNEL) assay

Apoptosis in human SG tissues was identified by TUNEL staining using an *in-situ* Cell Death Detection Kit (Roche, Germany). Numbers of apoptotic cells were quantified by digital microscopy (Olympus, Japan), and apoptotic indices were calculated using three random fields per sample.

### Cell culture

For human SG epithelial cell culture, a small portion of non-tumor bearing gland tissue was washed with HBSS containing 1% antibiotics, chopped with a pair of fine scissors for 7 min, enzymatically digested with 0.25% collagenase type B (2.5 mg/mL) and DNase I (1 mg/mL) with gentle shaking at 37°C for 30 minutes, filtered through a 70 μm cell strainer, and centrifuged at 1500 rpm for 3 min. Cells were plated on a culture dish in Keratinocyte serum-free medium (Gibco, USA) containing L-glutamine, 2.5 μg of EGF (epidermal growth factor), 0.09 mM CaCl_2_, and 1% antibiotics and incubated in a 5% CO_2_ atmosphere at 37°C.

### RNA interference

Small interfering RNAs (siRNAs) targeting ADIPOQ (Forward: 5′-CCA UGA CAC CAA CUG AUC AUU-3′, Reverse: 5′-UGA UCA GUU GGU GUC AUG GUU-3′) and scrambled siRNA (Forward 5′-CCU CGU GCC GUU CCA UCA GGU AGU U-3′, Reverse: 5′-CUA CCU GAU GGA ACG GCA CGA GGU U-3′) were purchased from Genolution (Seoul, Korea). SG cells were transfected with 40 nM of siRNA using G-fectin transfection reagent (Genolution) for 72 hr, and protein levels were assayed 72 hr after transfection.

### Proliferation assay

After treating SG cells with siRNA for 72 hr, cells were detached, and plated in a 96-well plate in 100 μL complete medium at a density of 4 × 10^3^ cells/well and cellular proliferations were measured at four time points (days 0, 1, 3, and 5) by CELLOMAX^™^ solution (PreCareGene, Korea). On each day, cells were treated with CELLOMAX^™^ solution and incubated for one hour at 37°C in 5% CO_2_. Absorbance was read at 450 nm with a microplate reader (Molecular Devices, USA).

### Senescence-associated β-galactosidase staining

SA-β-gal-positive cells were identified using a senescence β-galactosidase staining kit (Sigma Aldrich, USA). In brief, fixed SG cells were washed 3 times with PBS and incubated with a staining mixture in the absence of CO_2_ for 24 hr at 37°C. Blue-stained cells were visualized under a digital microscope, and senescent cells were counted in three random fields per slide using Image J (MD Anderson Center, USA).

### RNA isolation and real time PCR

Total RNA was isolated from human salivary gland epithelial cells using the RNeasy Mini kit (Qiagen, Germany), and cDNA was synthesized from total RNA using the Tetro cDNA synthesis kit (Bioline, USA). In brief, the cDNA reaction mixture was incubated for 30 min at 45°C, heated for 5 min at 85°C, and cooled to 4°C. Using cDNA as a template, real-time PCR was performed in 96-well plates (Applied Biosystems, USA) using SYBR green II Master Mix (Takara Bio Inc., Japan) in a StepOne unit (Applied Biosystems, USA) using the following program: 95°C for 20 s followed by 40 amplification cycles of 95°C for 5 s and 60°C for 20 s. The primers used were: IL-8 forward, 5′-TTT TGC CAA GGA GTG CTA AAG A-3′ and reverse, 5′-AAC CCT CTG CAC CCA GTT TTC-3′; p16 forward, 5′-CAA CGC ACC GAA TAG TTA CG-3′, and reverse, 5′-CAG CTC CTC AGC CAG GTC-3′; Adipoq forward, 5′-CTA TGA TGG CTC CAC TGG TA-3′ and reverse, 5′-GAG CAT AGC CTT GTC CTT CT-3′; AQP5 forward, 5′-ACT GGG TTT TCT GGG TAG GG -3′ and reverse, 5′-GTG GTC AGC TCC ATG GTC TT-3′; Amylase forward, 5′-ACA TGG GGC TGG AGG AGC CT-3′, and reverse, 5′-TGG TGG CCC AAC CCA ATC AT-3′ and β-actin (the endogenous control) forward, 5′-AGC TGT GCT ATG TTG CCC TG-3′, and reverse, 5′-AGG AAG CAA GGC TGG AAG AG-3′.

### Western blotting

Cells were homogenized in PRO-PREP^™^ protein extraction solution (iNtRON Biotechnology, Korea), incubated on ice for 30 min, centrifuged at 13,000 rpm for 10 min at 4°C, and supernatants were collected. Primary antibodies for ADIPOQ, amylase, AQP5, p16, p21, and GAPDH (all from Santa Cruz Biotechnology, 1:1000) were used. Goat anti-mouse IgG-HRP was used as the secondary antibody (Santa Cruz Biotechnology, 1:5000). Proteins were visualized using SuperSignal^™^ West Femto Maximum Sensitivity Substrate (Thermo Fisher Scientific, USA) using a ImageQunat^™^ LAS 4000 unit (GE Healthcare, USA).

## Supplementary Materials

Supplementary Figures

Supplementary Table 1

## References

[1] Mitterberger MC, Lechner S, Mattesich M, Zwerschke W. Adipogenic differentiation is impaired in replicative senescent human subcutaneous adipose-derived stromal/progenitor cells. J Gerontol A Biol Sci Med Sci. 2014; 69:13–24. 10.1093/gerona/glt043 23657974

[2] Choi JS, Park IS, Kim SK, Lim JY, Kim YM. Analysis of age-related changes in the functional morphologies of salivary glands in mice. Arch Oral Biol. 2013; 58:1635–42. 10.1016/j.archoralbio.2013.07.008 24112729

[3] De Wilde PC, Baak JP, van Houwelingen JC, Kater L, Slootweg PJ. Morphometric study of histological changes in sublabial salivary glands due to aging process. J Clin Pathol. 1986; 39:406–17. 10.1136/jcp.39.4.406 3700674 PMC499837

[4] Dayan D, Vered M, Paz T, Buchner A. Aging of human palatal salivary glands: a histomorphometric study. Exp Gerontol. 2000; 35:85–93. 10.1016/s0531-5565(99)00079-0 10705042

[5] Leehan KM, Pezant NP, Rasmussen A, Grundahl K, Moore JS, Radfar L, Lewis DM, Stone DU, Lessard CJ, Rhodus NL, Segal BM, Kaufman CE, Scofield RH, et al. Fatty infiltration of the minor salivary glands is a selective feature of aging but not Sjögren's syndrome. Autoimmunity. 2017; 50:451–7. 10.1080/08916934.2017.1385776 28988489 PMC5730459

[6] Kim SK. Changes in the secretory acinar cells of the rat parotid gland during aging. Anat Rec. 1984; 209:345–54. 10.1002/ar.1092090313 6465543

[7] Devireddy LR, Gazin C, Zhu X, Green MR. A cell-surface receptor for lipocalin 24p3 selectively mediates apoptosis and iron uptake. Cell. 2005; 123:1293–305. 10.1016/j.cell.2005.10.027 16377569

[8] Elisen MG, von dem Borne PA, Bouma BN, Meijers JC. Protein C inhibitor acts as a procoagulant by inhibiting the thrombomodulin-induced activation of protein C in human plasma. Blood. 1998; 91:1542–7. 10.1182/blood.V91.5.1542 9473218

[9] Komori R, Kobayashi T, Matsuo H, Kino K, Miyazawa H. Csn3 gene is regulated by all-trans retinoic acid during neural differentiation in mouse P19 cells. PLoS One. 2013; 8:e61938. 10.1371/journal.pone.0061938 23613978 PMC3629135

[10] Lee HK, Ji S, Park SJ, Choung HW, Choi Y, Lee HJ, Park SY, Park JC. Odontogenic Ameloblast-associated Protein (ODAM) Mediates Junctional Epithelium Attachment to Teeth via Integrin-ODAM-Rho Guanine Nucleotide Exchange Factor 5 (ARHGEF5)-RhoA Signaling. J Biol Chem. 2015; 290:14740–53. 10.1074/jbc.M115.648022 25911094 PMC4505539

[11] Ligtenberg AJ, Veerman EC, Nieuw Amerongen AV, Mollenhauer J. Salivary agglutinin/glycoprotein-340/DMBT1: a single molecule with variable composition and with different functions in infection, inflammation and cancer. Biol Chem. 2007; 388:1275–89. 10.1515/BC.2007.158 18020944

[12] Lu X, Xu Y, Zhao Y, Tao Q, Wu J. Silenced DMBT1 promotes nasal mucosa epithelial cell growth. Ann Hum Genet. 2018; 82:102–8. 10.1111/ahg.12230 29148567

[13] Okabe H, Satoh S, Furukawa Y, Kato T, Hasegawa S, Nakajima Y, Yamaoka Y, Nakamura Y. Involvement of PEG10 in human hepatocellular carcinogenesis through interaction with SIAH1. Cancer Res. 2003; 63:3043–8. 12810624

[14] Sohn JH, Lee YK, Han JS, Jeon YG, Kim JI, Choe SS, Kim SJ, Yoo HJ, Kim JB. Perilipin 1 (Plin1) deficiency promotes inflammatory responses in lean adipose tissue through lipid dysregulation. J Biol Chem. 2018; 293:13974–88. 10.1074/jbc.RA118.003541 30042231 PMC6130955

[15] Wang F, Chen S, Liu HB, Parent CA, Coulombe PA. Keratin 6 regulates collective keratinocyte migration by altering cell-cell and cell-matrix adhesion. J Cell Biol. 2018; 217:4314–30. 10.1083/jcb.201712130 30389720 PMC6279382

[16] Xu A, Sweeney G. Emerging role of autophagy in mediating widespread actions of ADIPOQ/adiponectin. Autophagy. 2015; 11:723–4. 10.1080/15548627.2015.1034418 25905437 PMC4502648

[17] Yamauchi T, Kamon J, Waki H, Terauchi Y, Kubota N, Hara K, Mori Y, Ide T, Murakami K, Tsuboyama-Kasaoka N, Ezaki O, Akanuma Y, Gavrilova O, et al. The fat-derived hormone adiponectin reverses insulin resistance associated with both lipoatrophy and obesity. Nat Med. 2001; 7:941–6. 10.1038/90984 11479627

[18] Yoon BR, Oh YJ, Kang SW, Lee EB, Lee WW. Role of SLC7A5 in Metabolic Reprogramming of Human Monocyte/Macrophage Immune Responses. Front Immunol. 2018; 9:53. 10.3389/fimmu.2018.00053 29422900 PMC5788887

[19] Zhang H, Huo M, Jia Y, Xu A. KRT6B, a key mediator of notch signaling in honokiol-induced human hepatoma cell apoptosis. Int J Clin Exp Med. 2015; 8:16880–9. 26629239 PMC4659127

[20] Zheng X, Geiger M, Ecke S, Bielek E, Donner P, Eberspächer U, Schleuning WD, Binder BR. Inhibition of acrosin by protein C inhibitor and localization of protein C inhibitor to spermatozoa. Am J Physiol. 1994; 267:C466–72. 10.1152/ajpcell.1994.267.2.C466 7521127

[21] Paton CM, Ntambi JM. Biochemical and physiological function of stearoyl-CoA desaturase. Am J Physiol Endocrinol Metab. 2009; 297:E28–37. 10.1152/ajpendo.90897.2008 19066317 PMC2711665

[22] McHeyzer-Williams M, Okitsu S, Wang N, McHeyzer-Williams L. Molecular programming of B cell memory. Nat Rev Immunol. 2011; 12:24–34. 10.1038/nri3128 22158414 PMC3947622

[23] Syrjänen S. Age-related changes in structure of labial minor salivary glands. Age Ageing. 1984; 13:159–65. 10.1093/ageing/13.3.159 6731173

[24] Enoki N, Kiyoshima T, Sakai T, Kobayashi I, Takahashi K, Terada Y, Sakai H. Age-dependent changes in cell proliferation and cell death in the periodontal tissue and the submandibular gland in mice: a comparison with other tissues and organs. J Mol Histol. 2007; 38:321–32. 10.1007/s10735-007-9105-6 17578672

[25] Kwon HK, Kim JM, Shin SC, Sung ES, Kim HS, Park GC, Cheon YI, Lee JC, Lee BJ. The mechanism of submandibular gland dysfunction after menopause may be associated with the ferroptosis. Aging (Albany NY). 2020; 12:21376–90. 10.18632/aging.103882 33159020 PMC7695378

[26] Knaś M, Maciejczyk M, Daniszewska I, Klimiuk A, Matczuk J, Kołodziej U, Waszkiel D, Ładny JR, Żendzian-Piotrowska M, Zalewska A. Oxidative Damage to the Salivary Glands of Rats with Streptozotocin-Induced Diabetes-Temporal Study: Oxidative Stress and Diabetic Salivary Glands. J Diabetes Res. 2016; 2016:4583742. 10.1155/2016/4583742 27478848 PMC4961808

[27] Solinas G, Karin M. JNK1 and IKKbeta: molecular links between obesity and metabolic dysfunction. FASEB J. 2010; 24:2596–611. 10.1096/fj.09-151340 20371626

[28] Rolo AP, Teodoro JS, Palmeira CM. Role of oxidative stress in the pathogenesis of nonalcoholic steatohepatitis. Free Radic Biol Med. 2012; 52:59–69. 10.1016/j.freeradbiomed.2011.10.003 22064361

[29] Ding C, Li L, Su YC, Xiang RL, Cong X, Yu HK, Li SL, Wu LL, Yu GY. Adiponectin increases secretion of rat submandibular gland via adiponectin receptors-mediated AMPK signaling. PLoS One. 2013; 8:e63878. 10.1371/journal.pone.0063878 23667684 PMC3646765

[30] Kadowaki T, Yamauchi T. Adiponectin and adiponectin receptors. Endocr Rev. 2005; 26:439–51. 10.1210/er.2005-0005 15897298

[31] Shibata R, Ouchi N, Ito M, Kihara S, Shiojima I, Pimentel DR, Kumada M, Sato K, Schiekofer S, Ohashi K, Funahashi T, Colucci WS, Walsh K. Adiponectin-mediated modulation of hypertrophic signals in the heart. Nat Med. 2004; 10:1384–9. 10.1038/nm1137 15558058 PMC2828675

[32] Shibata R, Sato K, Pimentel DR, Takemura Y, Kihara S, Ohashi K, Funahashi T, Ouchi N, Walsh K. Adiponectin protects against myocardial ischemia-reperfusion injury through AMPK- and COX-2-dependent mechanisms. Nat Med. 2005; 11:1096–103. 10.1038/nm1295 16155579 PMC2828682

[33] Shibata R, Izumiya Y, Sato K, Papanicolaou K, Kihara S, Colucci WS, Sam F, Ouchi N, Walsh K. Adiponectin protects against the development of systolic dysfunction following myocardial infarction. J Mol Cell Cardiol. 2007; 42:1065–74. 10.1016/j.yjmcc.2007.03.808 17499764 PMC1987393

[34] Staiger K, Stefan N, Staiger H, Brendel MD, Brandhorst D, Bretzel RG, Machicao F, Kellerer M, Stumvoll M, Fritsche A, Häring HU. Adiponectin is functionally active in human islets but does not affect insulin secretory function or beta-cell lipoapoptosis. J Clin Endocrinol Metab. 2005; 90:6707–13. 10.1210/jc.2005-0467 16204361

[35] Gröschl M, Rauh M, Wagner R, Neuhuber W, Metzler M, Tamgüney G, Zenk J, Schoof E, Dörr HG, Blum WF, Rascher W, Dötsch J. Identification of leptin in human saliva. J Clin Endocrinol Metab. 2001; 86:5234–9. 10.1210/jcem.86.11.7998 11701683

[36] Katsiougiannis S, Kapsogeorgou EK, Manoussakis MN, Skopouli FN. Salivary gland epithelial cells: a new source of the immunoregulatory hormone adiponectin. Arthritis Rheum. 2006; 54:2295–9. 10.1002/art.21944 16802369

[37] Katsiougiannis S, Tenta R, Skopouli FN. Activation of AMP-activated protein kinase by adiponectin rescues salivary gland epithelial cells from spontaneous and interferon-gamma-induced apoptosis. Arthritis Rheum. 2010; 62:414–9. 10.1002/art.27239 20112400

[38] Choi HM, Doss HM, Kim KS. Multifaceted Physiological Roles of Adiponectin in Inflammation and Diseases. Int J Mol Sci. 2020; 21:1219. 10.3390/ijms21041219 32059381 PMC7072842

[39] Miyagi Y, Kondo Y, Kusuda Y, Hori Y, Yamazaki S, Munemasa T, Mukaibo T, Masaki C, Hosokawa R. Submandibular gland-specific inflammaging-induced hyposalivation in the male senescence-accelerated mouse prone -1 line (SAM-P1). Biogerontology. 2019; 20:421–32. 10.1007/s10522-019-09797-3 30684147

[40] Franceschi C, Campisi J. Chronic inflammation (inflammaging) and its potential contribution to age-associated diseases. J Gerontol A Biol Sci Med Sci. 2014 (Suppl 1); 69:S4–9. 10.1093/gerona/glu057 24833586

[41] Kim JM, Choi ME, Kim SK, Kim JW, Kim YM, Choi JS. Keratinocyte Growth Factor-1 Protects Radioiodine-Induced Salivary Gland Dysfunction in Mice. Int J Environ Res Public Health. 2020; 17:6322. 10.3390/ijerph17176322 32878050 PMC7503708

