# Supplementary material for: Adiponectin is associated with inflammaging and age-related salivary gland lipid accumulation
Source: Aging (Albany NY). 2023 Mar 27;15(6):1840–58. doi: 10.18632/aging.204618 (PMC10085617; doi:10.18632/aging.204618)
Supplement: Supplementary Figures [file aging-15-204618-s001.pdf]

## SUPPLEMENTARY FIGURES

| Gene_Symbol | Gene_ID | mRNA Accession | Fold Change Aged/Adult (PG) |
|---|---|---|---|
| IGHM | 3507 | BC011857 | 6.968879 |
| LOC101927120 | 101927120 | NR_110136 | 2.908335 |
| SLC7A5 | 8140 | NM_003486 | 2.841554 |
| KRTAP5-2 | 440021 | NM_001004325 | 2.203520 |
| SNORD37 | 26812 | NR_002602 | 2.157255 |
| KRT6B | 3854 | NM_005555 | 2.069454 |
| SERPINA5 | 5104 | NM_000624 | 2.015452 |
| LOC105375217 | 105375217 | XR_927147 | 1.925442 |
| MYH1 | 4619 | NM_005963 | 1.910244 |
| VTRNA1-1 | 56664 | NR_026703 | 1.909657 |
| GOLGA6L7P | 728310 | NR_047567 | 1.845630 |
| LTF | 4057 | NM_001199149 | 1.815881 |
| MIR548AN | 100616144 | NR_039765 | 1.809482 |
| GH2 | 2689 | NM_002059 | 1.775243 |
| ZNF708 | 7562 | NM_001297560 | 1.749792 |
| LOC105375044 | 105375044 | XR_926767 | 1.689906 |
| CACNA1D | 776 | NM_000720 | 1.635336 |
| LCE3E | 353145 | NM_178435 | 1.627860 |
| ZMAT3 | 64393 | NM_022470 | 1.597397 |
| MIR520E | 574461 | NR_030183 | 1.557508 |
| MIR891A | 100126341 | NR_030581 | 1.548770 |
| LOC100507494 | 100507494 | AK090481 | 1.539391 |
| SNORD115-45 | 100036566 | NR_003498 | 1.537014 |
| LRRC3C | 100505591 | NM_001195545 | 1.526499 |
| MAPT-IT1 | 100130148 | ENST00000626958 | 1.513355 |
| LOC105376527 | 105376527 | XR_930988 | 1.506562 |
| LINC01524 | 101927700 | NR_110038 | -1.501124 |
| LRP2 | 4036 | NM_004525 | -1.503005 |
| IQCF2 | 389123 | NM_203424 | -1.508920 |
| FRMD8P1 | 83957 | NR_033742 | -1.510207 |
| AVPR2 | 554 | NM_000054 | -1.522772 |
| LINC01015 | 100507362 | NR_037179 | -1.523004 |
| STX19 | 415117 | NM_001001850 | -1.535952 |
| OR4S2 | 219431 | NM_001004059 | -1.546214 |
| LOC102724104 | 102724104 | XR_937125 | -1.594341 |
| USP17L22 | 100287513 | NM_001256863 | -1.623754 |
| MIR4645 | 100616285 | NR_039788 | -1.632908 |
| MIR4730 | 100616359 | NR_039883 | -1.633304 |
| NEBL-AS1 | 100128511 | NR_046283 | -1.651908 |
| LOC105376165 | 105376165 | XR_930152 | -1.658585 |
| LOC105379082 | 105379082 | XR_948567 | -1.678102 |
| TCP10L2 | 401285 | XM_011535822 | -1.687725 |
| MCOLN3 | 55283 | NM_001253693 | -1.710170 |
| CFTR | 1080 | NM_000492 | -1.748196 |
| CCDC122 | 160857 | NM_144974 | -1.756902 |
| LOC105374086 | 105374086 | XM_011536382 | -1.760010 |
| TAC1 | 6863 | NM_003182 | -1.878416 |
| OR4C16 | 219428 | NM_001004701 | -1.911966 |
| TMEM52B | 120939 | NM_001079815 | -1.945723 |
| SERHL | 94009 | NR_027786 | -2.003061 |
| PPBP | 5473 | NM_002704 | -2.012441 |
| MIR516A1 | 574498 | NR_030220 | -2.021020 |
| GK | 2710 | NM_000167 | -2.083833 |
| LINC01139 | 339535 | NR_015407 | -2.090860 |
| SCD | 6319 | NM_005063 | -2.145316 |
| RNU12 | 267010 | NR_029422 | -2.211497 |
| CES1P1 | 51716 | ENST00000421606 | -2.259019 |

**Supplementary Figure 1. List of DEGs between adult and aged parotid glands.** List of differentially expressed genes (DEGs) between adult and aged parotid gland (based on at least a 1.5-fold change and $p$ value < 0.05).

| Gene_Symbol | Gene_ID | mRNA Accession | Fold Change Aged/Adult (SMG) |
|---|---|---|---|
| CSN3 | 1448 | NM_005212 | 3.877379 |
| ODAM | 54959 | NM_017855 | 3.621289 |
| ZCWPW2 | 152098 | AB087877 | 3.485096 |
| ADIPOQ | 9370 | NM_001177800 | 3.281488 |
| PLIN1 | 5346 | NM_001145311 | 2.753045 |
| TRDJ4 | 28519 | OTTHUMT00000410933 | 2.563580 |
| PEG10 | 23089 | NM_001040152 | 2.513301 |
| DMBT1 | 1755 | NM_004406 | 2.477414 |
| LCN2 | 3934 | NM_005564 | 2.469396 |
| RPL23AP7 | 118433 | NR_000029 | 2.445967 |
| CXCL17 | 284340 | NM_198477 | 2.402523 |
| MIR378E | 100616498 | NR_039609 | 2.359912 |
| H3F3B | 3021 | NM_005324 | 2.209932 |
| GLP1R | 2740 | NM_002062 | 2.207696 |
| C10orf107 | 219621 | NM_173554 | 2.138469 |
| MIR4739 | 100616170 | NR_039893 | 2.130492 |
| NPIPB11 | 728888 | XM_011546005 | 2.109301 |
| LOC644135 | 644135 | XR_943016 | 2.056472 |
| LRRC32 | 2615 | NM_001128922 | 2.033439 |
| SLC13A2 | 9058 | NM_001145975 | 2.021024 |
| LAMA2 | 3908 | NM_000426 | 2.014116 |
| ITM2A | 9452 | NM_001171581 | 1.989942 |
| MMP7 | 4316 | NM_002423 | 1.968020 |
| HSPB6 | 126393 | NM_144617 | 1.908638 |
| HIST2H2BE | 8349 | NM_003528 | 1.908203 |
| LOC729162 | 729162 | XR_950549 | 1.884894 |
| GFRA3 | 2676 | NM_001496 | 1.848942 |
| FAM66A | 100133172 | NR_026789 | 1.847736 |
| EBF1 | 1879 | NM_001290360 | 1.788372 |
| MIR29C | 407026 | NR_029832 | 1.784049 |
| MEIS3P1 | 4213 | NR_002211 | 1.783632 |
| NAGPA-AS1 | 100507589 | NR_038913 | 1.783336 |
| MIR4473 | 100616229 | NR_039684 | 1.771455 |
| PNRC2 | 55629 | NM_017761 | 1.763217 |
| TLR7 | 51284 | NM_016562 | 1.746700 |
| FMOD | 2331 | NM_002023 | 1.744332 |
| SPANXN5 | 494197 | NM_001009616 | 1.742244 |
| GPNMB | 10457 | NM_001005340 | 1.688689 |
| NXF5 | 55998 | NM_032946 | 1.679810 |
| ANPEP | 290 | NM_001150 | 1.679531 |
| LOC105376093 | 105376093 | XR_929954 | 1.652129 |
| IRX6 | 79190 | NM_024335 | 1.649560 |
| ANKRD10 | 55608 | NR_104586 | 1.646286 |
| HNRNPC | 3183 | NM_001077442 | 1.638772 |
| ZNF284 | 342909 | NM_001037813 | 1.633540 |
| LOC105373730 | 105373730 | XR_923551 | 1.622980 |
| MIR4494 | 100616478 | NR_039715 | 1.616950 |
| MGAM2 | 93432 | NM_001293626 | 1.608772 |
| LINC01506 | 101927015 | NR_109769 | 1.591292 |
| MSC-AS1 | 100132891 | NR_033651 | 1.586123 |
| LY86 | 9450 | NM_004271 | 1.581566 |
| CGB8 | 94115 | NM_033183 | 1.574725 |
| SELPLG | 6404 | NM_001206609 | 1.571848 |
| MUC20 | 200958 | NM_001282506 | 1.566180 |
| LOC102723505 | 102723505 | NR_110876 | 1.560612 |
| HLA-DQB2 | 3120 | NM_001198858 | 1.549792 |
| IGHV1-69 | 28461 | OTTHUMT00000324207 | 1.548153 |
| IL17RD | 54756 | NM_017563 | 1.545380 |
| ITGA10 | 8515 | NM_001303040 | 1.542583 |
| MIR765 | 768220 | NR_030527 | 1.537287 |
| ATP1B4 | 23439 | NM_001142447 | 1.530152 |
| SNORD115-32 | 100033806 | NR_003347 | 1.525332 |
| MCF2 | 4168 | NM_001099855 | 1.522990 |
| PLK2 | 10769 | NM_001252226 | 1.522093 |
| LYSMD4 | 145748 | NM_001284417 | 1.521513 |
| EYA1 | 2138 | NM_000503 | 1.519507 |
| C5orf46 | 389336 | NM_206966 | 1.518773 |
| MCF2L-AS1 | 100289410 | NR_034002 | 1.513757 |
| PLPP1 | 8611 | NM_003711 | 1.513729 |
| MIR323A | 442897 | NR_029890 | 1.506012 |
| BARX2 | 8538 | NM_003658 | 1.505732 |
| FUS | 2521 | NM_001170634 | 1.500209 |
| C5orf60 | 285679 | AK093042 | -1.503174 |
| LOC105374261 | 105374261 | XR_924809 | -1.510150 |
| MIR4318 | 100422857 | NR_036202 | -1.511169 |
| LOC105370642 | 105370642 | XR_915933 | -1.525863 |
| SCX | 642658 | NM_001080514 | -1.542630 |
| LINC00398 | 100874158 | NR_047012 | -1.562614 |
| RPL23P8 | 222901 | NR_026673 | -1.569134 |
| MIR3691 | 100500900 | NR_037462 | -1.604544 |
| OR10AD1 | 121275 | NM_001004134 | -1.606547 |
| MTRNR2L5 | 100463289 | NM_001190478 | -1.667533 |
| LOC101929715 | 101929715 | BC111372 | -1.688932 |
| LOC101929305 | 101929305 | XR_241170 | -1.690197 |
| SLC9B1P3 | 100421008 | OTTHUMT00000047660 | -1.692214 |
| SPANXC | 64663 | uc004fbk.3 | -1.694244 |
| SHISA2 | 387914 | NM_001007538 | -1.723789 |
| FAM171B | 165215 | NM_177454 | -1.736445 |
| KRT35 | 3886 | NM_002280 | -1.900736 |
| LOC105369381 | 105369381 | XR_913289 | -2.006661 |
| LOC728093 | 728093 | XR_948446 | -2.011749 |
| LOC101927120 | 101927120 | NR_110136 | -2.023709 |
| MUC5B | 727897 | NM_002458 | -2.038081 |
| LINC01372 | 101929736 | NR_108104 | -2.073445 |
| MIR3689E | 100616460 | NR_039695 | -2.123141 |
| MT1L | 4500 | NR_001447 | -2.145291 |
| MIR516A1 | 574498 | NR_030220 | -2.179300 |
| SCGB1C2 | 653486 | NM_001097610 | -4.843478 |
| C6orf58 | 352999 | NM_001010905 | -5.904671 |

**Supplementary Figure 2. List of DEGs between adult and aged submandibular glands.** List of differentially expressed genes (DEGs) between adult and aged submandibular gland (based on at least a 1.5-fold change and $p$ value < 0.05).

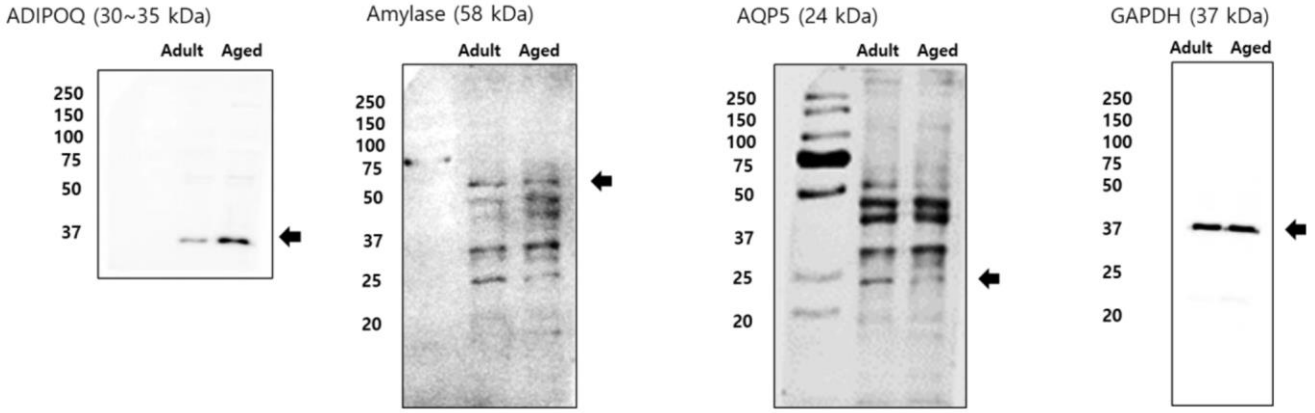


**Supplementary Figure 3. Uncropped western blots of Figure 5F.**

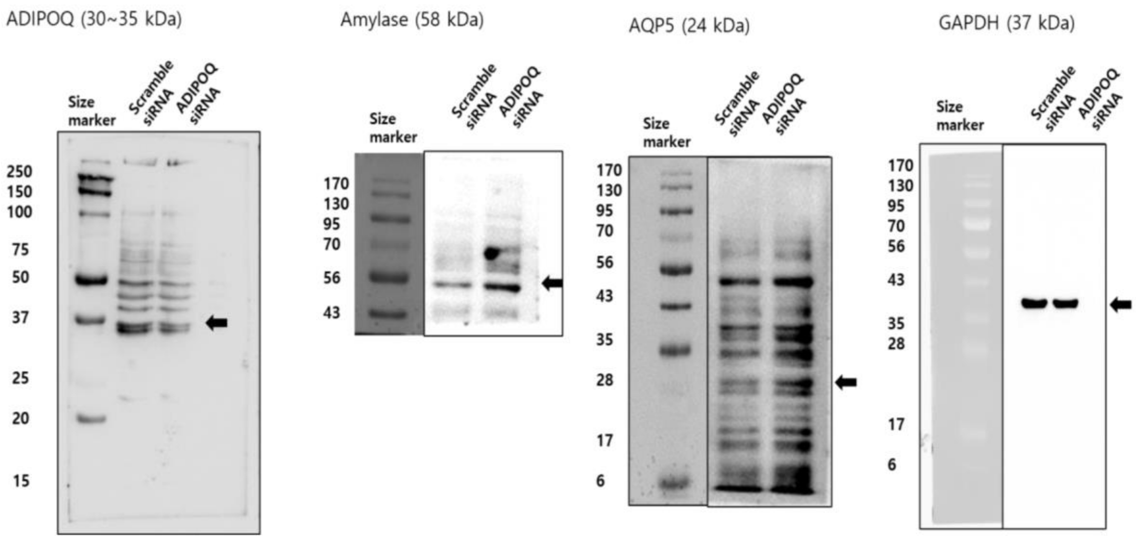


**Supplementary Figure 4. Uncropped western blots of Figure 6F.**

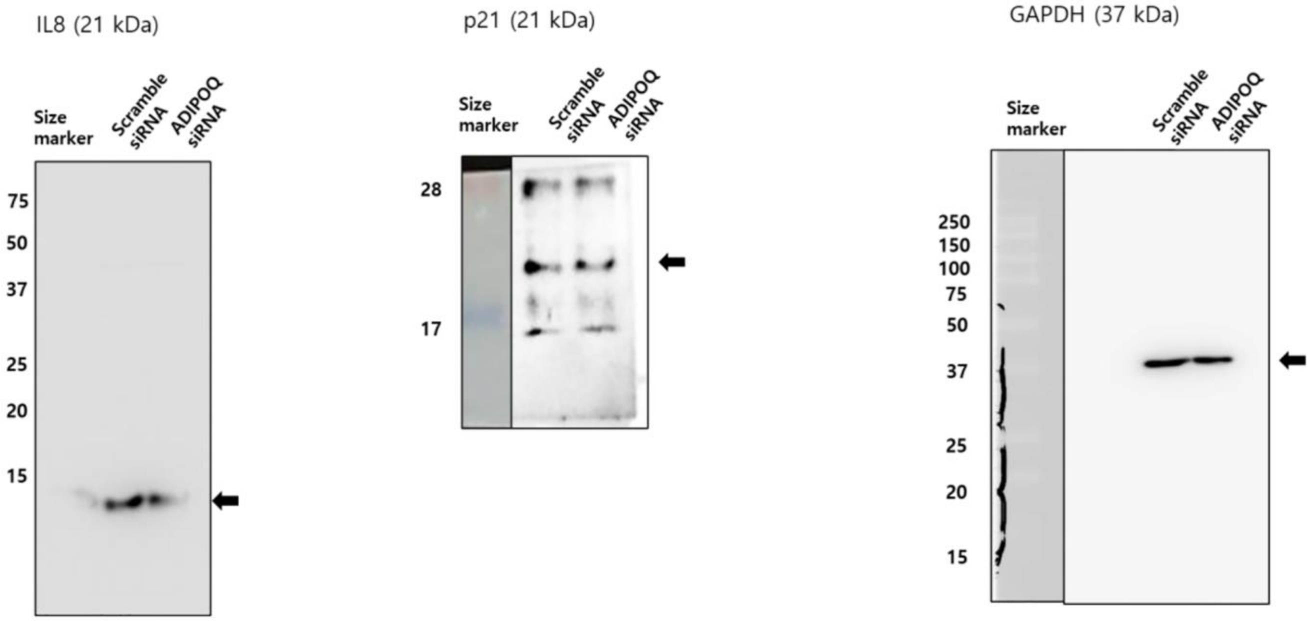


**Supplementary Figure 5. Uncropped western blots of Figure 7C.**

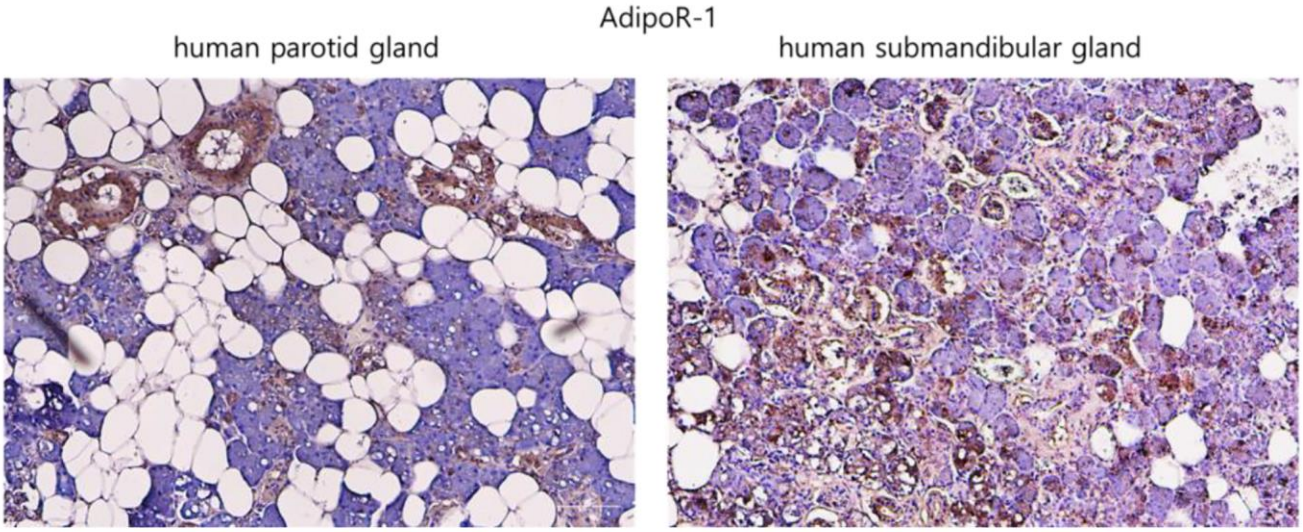


**Supplementary Figure 6. The expression of AdipoR1 in human salivary gland tissues.**
