# Supplementary material for: Adiponectin is associated with inflammaging and age-related salivary gland lipid accumulation
Source: Aging (Albany NY). 2023 Mar 27;15(6):1840–58. doi: 10.18632/aging.204618 (PMC10085617; doi:10.18632/aging.204618)
Supplement: Supplementary Table 1 [file aging-15-204618-s002.pdf]

SUPPLEMENTARY TABLE

Supplementary Table 1. The information of patients.

Tissue	Category	Patient No.	Age	Sex	BMI
PG	Adult	1	23	F	19.4
		2	27	F	19.0
		3	36	M	31.9
		Mean ± SD	28.6 ± 6.6	−	23.4 ± 7.3
	Aged	1	77	M	22.0
		2	84	M	25.8
		3	85	F	21.7
		Mean ± SD	82.0 ± 4.3	−	23.2 ± 2.2
SMG	Adult	1	43	M	25.6
		2	42	M	20.3
		3	45	F	22.4
		Mean ± SD	43.3 ± 1.5	−	22.8 ± 2.6
	Aged	1	86	M	23.0
		2	93	M	20.0
		3	85	F	22.4
		Mean ± SD	88.0 ± 4.3	−	21.8 ± 1.5
